# Identification of NAD(P)H Quinone Oxidoreductase Activity in Azoreductases from *P. aeruginosa*: Azoreductases and NAD(P)H Quinone Oxidoreductases Belong to the Same FMN-Dependent Superfamily of Enzymes

**DOI:** 10.1371/journal.pone.0098551

**Published:** 2014-06-10

**Authors:** Ali Ryan, Elise Kaplan, Jean-Christophe Nebel, Elena Polycarpou, Vincenzo Crescente, Edward Lowe, Gail M. Preston, Edith Sim

**Affiliations:** 1 Department of Pharmacology, University of Oxford, Oxford, United Kingdom; 2 Faculty of Science, Engineering and Computing, Kingston University, Kingston upon Thames, United Kingdom; 3 Laboratory of Molecular Biophysics, Biochemistry Department, University of Oxford, Oxford, United Kingdom; 4 Department of Plant Sciences, University of Oxford, Oxford, United Kingdom; Instituto de Biociencias - Universidade de São Paulo, Brazil

## Abstract

Water soluble quinones are a group of cytotoxic anti-bacterial compounds that are secreted by many species of plants, invertebrates, fungi and bacteria. Studies in a number of species have shown the importance of quinones in response to pathogenic bacteria of the genus *Pseudomonas*. Two electron reduction is an important mechanism of quinone detoxification as it generates the less toxic quinol. In most organisms this reaction is carried out by a group of flavoenzymes known as NAD(P)H quinone oxidoreductases. Azoreductases have previously been separate from this group, however using azoreductases from *Pseudomonas aeruginosa* we show that they can rapidly reduce quinones. Azoreductases from the same organism are also shown to have distinct substrate specificity profiles allowing them to reduce a wide range of quinones. The azoreductase family is also shown to be more extensive than originally thought, due to the large sequence divergence amongst its members. As both NAD(P)H quinone oxidoreductases and azoreductases have related reaction mechanisms it is proposed that they form an enzyme superfamily. The ubiquitous and diverse nature of azoreductases alongside their broad substrate specificity, indicates they play a wide role in cellular survival under adverse conditions.

## Introduction

Quinones are best known for playing an important role in the electron transport chain of most organisms either in the form of ubiquinones or menaquinones. The quinones involved in the electron transport chain are hydrophobic and lipid soluble. In contrast, water soluble quinones are cytotoxic.

The cytotoxicity of water soluble quinones stems from two primary mechanisms [1], firstly their ability to covalently modify DNA and proteins, secondly their ability to undergo redox cycling.

Water soluble quinones are used as defence mechanisms by a range of organisms. Water soluble quinones are produced as part of the innate immune response of invertebrates via the action of pro-phenoloxidase [2]. Plants and fungi use a functionally related family of enzymes called polyphenol oxidases (PPOs) to generate quinones [3]. The generation of quinones by PPO in plants and invertebrates leads to melanisation at the point of infection [4]. Some plants also secrete water soluble quinones into the soil to inhibit the growth of competing plants e.g. juglone from black walnut trees [5]. Bacteria themselves produce quinones as antibiotics e.g. doxorubicin from *Streptomyces peucetius* [6]. Thus it is important for bacteria to be able to detoxify these cytotoxic compounds.

**Figure 1:**
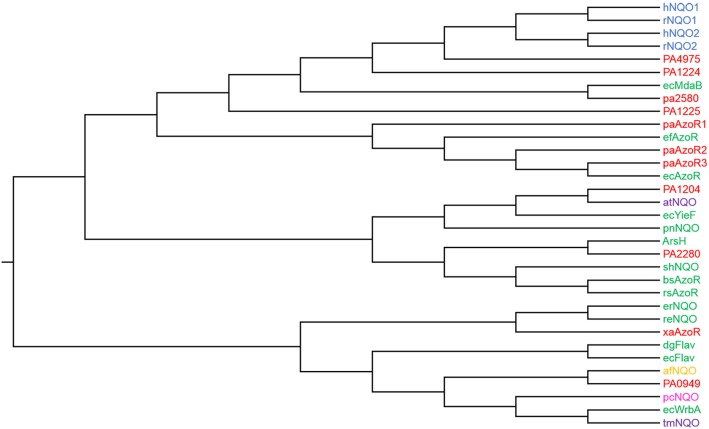
Phylogenetic tree illustrating relationships between known and suspected azoreductases from *P. aeruginosa* PAO1. An unrooted bootstrap consensus maximum parsimony tree was generated using 500 replicates in Mega 6 [78]. This tree was based upon a sequence alignment of 33 proteins using Muscle [79]. The tree was then rooted so that it is consistent with three previously published trees [74], [80], [81]. Enzymes in red are from *P. aeruginosa*, those in green are other bacterial enzymes, those in blue are mammalian enzymes, those in purple are plant enzymes, those in pink are fungal enzymes and those in yellow are archeal. PA0949, PA1204, PA1224, PA1225, PA2280, PA2580, and PA4975 are proteins from *P. aeruginosa*. ecAzoR, bsAzoR, efAzoR and rsAzoR are azoreductases from *E. coli*, *Bacillus subtilis*, *Enterococcus faecalus* and *Rhodobacter sphaeroides*. hNQO1 hNQO2, rNQO1 and rNQO2 are human and rat azoreductases. xaAzoR is a flavin independent azoreductase from *X. azovorans*. ecMdaB, ecYieF and ecWrbA are NAD(P)H quinone oxidoreductases from *E. coli*. afNQO, pnNQO, tmNQO, pcNQO and atNQO are NAD(P)H quinone oxidoreductases from *Archaeoglobus fulgidus*, *Paracoccus denitrificans*, *Triticum monococcum*, *Phanerochaete chrysosporium* and *Arabidopsis thaliana* respectively. ArsH is an azoreductase from *Sinorhizobium meliloti*. dgFlav and ecFlav are flavodoxins from *Desulfovibrio gigas* and *E. coli* respectively. shNQO, reNQO and erNQO are uncharacterised proteins from *Staphylococcus haemolyticus*, *Ralstonia eutropha* and *Erwinia chrysanthemi.*

One of the primary mechanisms of quinone detoxification is their two electron reduction to the more stable and less mutagenic quinol [7]. Two electron reduction reactions of quinones are carried out by a group of enzymes known as NAD(P)H quinone oxidoreductases. Azoreductases are a related but previously thought to be distinct group of NAD(P)H dependent flavoenzymes that have been identified in a number of species from bacteria [8]–[10], to mammals [11] (Fig. 1). Although azoreductases are constitutively expressed in a range of bacteria [10], [12], their physiological role remains unclear. Azoreductases are able to reduce a variety of substrate classes and some have been shown to reduce quinones however they have been tested against only a limited range of these substrates [8], [13].

Azoreduction has been shown to proceed via a bi-bi ping pong mechanism [14]. An X-ray crystallographic study into the mechanism of azoreduction by these enzymes suggested that the azo substrate must tautomerise to a hydrazone conformation prior to reduction [15]. In this mechanism reduction occurs via hydride transfer from the flavin group of the enzyme to a quinoneimine formed via tautomerisation of the azo bond. This mechanism therefore suggests that hydride transfer would also be possible to a quinone substrate [16]. Results from studies into reduction of quinones by a human azoreductase support this hypothesis by showing that that reduction occurs via an obligate two-electron reduction [17], [18] rather than via one-electron transfer as occurs in NADH dehydrogenases”.

*P. aeruginosa* is known to be a highly versatile pathogen, able to infect a range of hosts including invertebrates [19], plants and mammals [20]. Three azoreductases, called paAzoR1–3, have been characterised in *P. aeruginosa* [15], [21]. Preliminary data suggest that paAzoR1 from *P. aeruginosa* plays an important role in mammalian infection by the bacterium [22]. In light of the importance of quinones in the response of plants [23] and invertebrates [24] to infection by pathogenic Pseudomonads, azoreductases may also play a vital role in infection of other organisms via detoxification of their toxic quinones. We have therefore carried out a systematic study of the substrate specificity profiles of three recombinant azoreductases from *P. aeruginosa*. We also predict that the azoreductase family is much more extensive than originally thought. This data not only improves our understanding of the possible role of azoreductases in host/pathogen interactions but, also helps shed light on genomic data.

## Experimental Procedures

### Bioinformatics

The flow-diagram for the identification of azoreductase homologues in *P. aeruginosa* is provided in Fig. S1. An extensive literature review was performed via Google Scholar (search term: azoreductase) to identify proteins that have been characterised as able to reduce azo compounds. Non-redundant protein sequence database search was performed via Blastp [25] to identify enzymes related to these azo reducing proteins in the *P. aeruginosa* PAO1 genome [26]. Sequences of protein homologues should align well to at least 80% of the search sequence. In parallel proteins with a similar overall fold to paAzoR1 (Pfam families: flavodoxin_2 and FMN_red) were identified via the “3D similarity” tool within the PDB [27]. Literature searches were then performed on the proteins identified to have the same overall fold as paAzoR1, to determine which have a characterised quinone reductase activity. Blastp searches were then performed using the NAD(P)H quinone oxidoreductases identified to search for homologues in the *P. aeruginosa* PAO1 genome. Three previously distinct protein families (ArsH (named for arsenic resistance), tryptophan repressor binding protein A (WrbA) and modulator of drug activity B (MdaB)) were identified as putative azoreductases via these searches. The original search terms were thus expanded to include these families in order to help identify characterised proteins more closely related to the putative azoreductases in *P. aeruginosa*. All sequence similarities quoted in the text are based upon pairwise Blastp alignments. Once the azoreductase homologues had been identified in *P. aeruginosa* PAO1, their sequences were used to perform searches in other *Pseudomonas* genomes using Blastp.

Flavin-independent azoreductases such as AzoB from *Xenophilus azovorans* [28], were excluded from this analysis. This decision was made because they are a structurally unrelated (Pfam family: NAD_binding_10) family of monomeric enzymes. FMN independent azoreductases use an unrelated mechanism to reduce azo substrates that requires them to bind NAD(P)H which directly reduces the azo dye bypassing the intermediate steps of FMN reduction and oxidation.

### Enzymatic Assays

**Figure 2:**
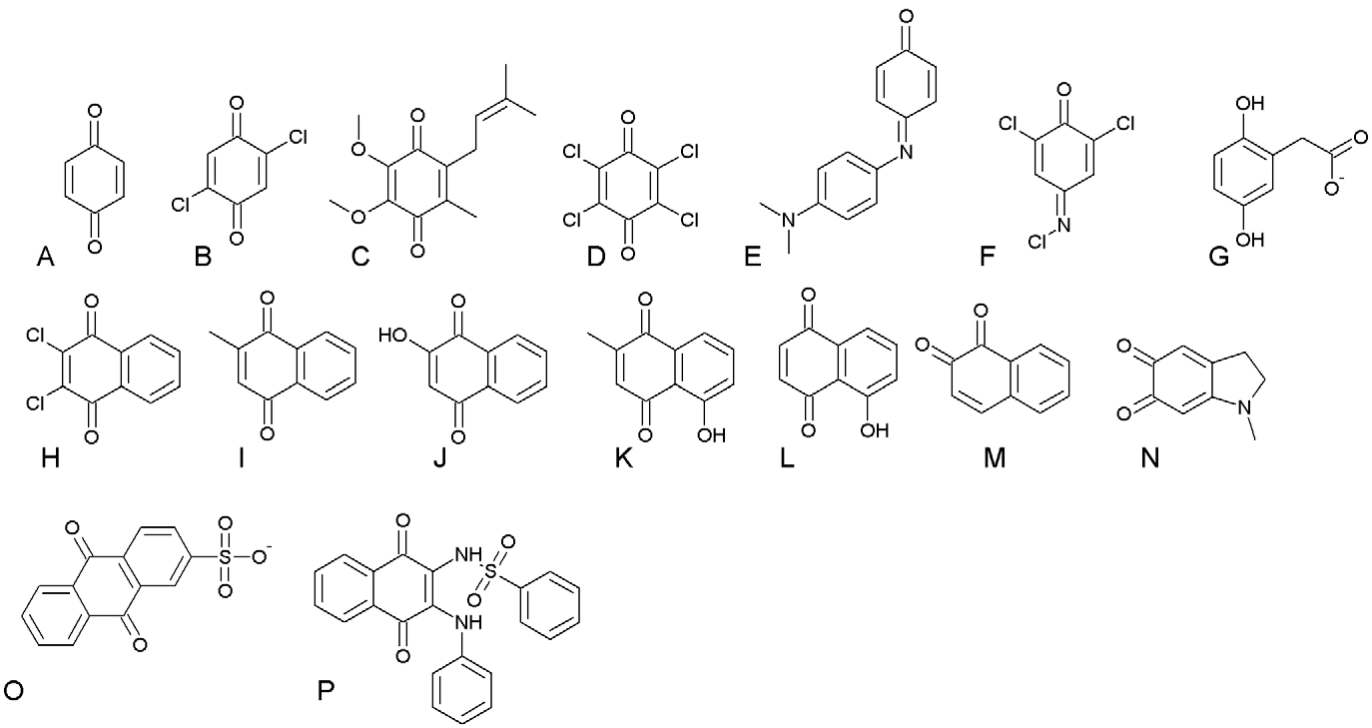
Structures of the quinone substrates tested in this manuscript. (A) Bzq, (B) Dcb, (C) UQ1, (D) Tco, (E) Phb, (F) Ibc, (G) Hom, (H) Dcn, (I) Men, (J) Law, (K) Plu, (L) Jug, (M) Onq, (N) Ade, (O) AQN, (P) Co7.

All reagents were obtained from Sigma-Aldrich. The pure recombinant proteins paAzoR1 (PA0785), paAzoR2 (PA1962) and paAzoR3 (PA3223) were expressed and purified as described previously [15], [21]. Rates of quinone reduction were obtained by monitoring the absorbance at 340 nm for oxidation of either NADPH (paAzoR1) or NADH (paAzoR2 and paAzoR3). Reactions were carried out in a 100 µL reaction volume in UV transparent 96-well plates (Greiner) and monitored using a Flurostar Omega plate reader (BMG labtech). Reaction mixtures were setup as follows: 50 µM quinone, 500 µM NAD(P)H and between 10 µg and 0.1 µg enzyme in 20 mM Tris-HCl pH 8, 100 mM NaCl, 5% (v/v) DMSO. Controls were performed in the absence of enzyme and all reactions were initiated by addition of 95 µL of enzyme and NAD(P)H to 5 µL quinone. Rates were determined by fitting the change in OD_340_ over the first five minutes. The quinones tested were as follows (Fig. 2): Coenzyme Q_1_ (UQ1), Phenol blue (Phb), Benzoquinone (Bzq), Juglone (Jug), Lawsone (Law), Menadione (Men), Plumbagin (Plu), Adrenochrome (Adr), 1,2-napthoquinone (Onq), 2,3-dichloro-1,4-napthoquinone (Dcn), 2,5-dichlorobenzoquinone (Dcb), 2,3,5,6-tetrachloro-1,4-benzoquinone (Tcq), 2,6-dichloroquinone-4-chloromide (Ibc) and anthraquinone-2-sulphonate (AQN). All quinones were prepared as 20 mM stocks dissolved in DMSO.

### Structure Determination

Solutions of paAzoR1 with either UQ1 or AQN were made up as follows: 23 mg.mL^−1^ paAzoR1 in 20 mM Tris-HCl pH 8, 2 mM AQN/UQ1 and incubated for one hour at room temperature before being washed with 20 mM Tris-HCl pH 8 to remove DMSO. Crystals grew isomorphously under the same conditions described previously [21]. Data for paAzoR1-AQN were collected at the Diamond Light Source (Oxon, UK) on beamline I03 using a Quantum ADSC detector. Data for paAzoR1-UQ1 were collected at the ESRF (Grenoble, France) on beamline ID23.2 with a MAR225 detector. paAzoR1-AQN and paAzoR1-UQ1 data processing were carried out in xia2 [29] using XDS [30], and SCALA [31]. The structures were solved via molecular replacement using Phaser [32], and the structure of paAzoR1 stripped of its ligands (PDB: 2V9C [21]) as the search model. The atomic models were rebuilt in Coot [33] and refined using Refmac [34] and PHENIX [35]. Restraints for AQN and UQ1 were generated in eLBOW [36]. Model validation was performed with MolProbity [37]. Data collection and refinement statistics are shown in Table 1 and the structures were deposited in the protein databank (PDB ID: 4N65 and 4N9Q).

**Table 1:** Processing and refinement statistics for paAzoR1 complexed with AQN and UQ1.

Structure	paAzoR1-UQ1	paAzoR1-AQN
Space Group	P3_1_ 2 1	P3_1_ 2 1
α,β,γ (°)	90, 90, 120	90, 90, 120
*a, b, c* (Å)	82.40, 82.40, 108.47	81.89, 81.89, 109.32
**Processing Statistics**		
Resolution Range (Å)	43.18–2.0	43.3–1.8
Unique reflections^a^	26,575 (2,687)	40,071 (2,713)
*R*_merge_	0.053 (0.462)	0.061 (0.351)
<I/σ(I)>^a^	17.1 (2.1)	15.5(3.2)
Completeness %^a^	90.7 (64.0)	94.9 (66.0)
Multiplicity^a^	4.7 (2.5)	5.1 (3.8)
**Refinement Statistics**		
*R*_work_ %	18.51	16.24
*R*_free_ %	23.57	18.51
RMS bond angle (°)	0.714	1.3
RMS bond length (Å)	0.003	0.011
**Ramachandran Statistics** b		
Preferred region %	98.5	98.5
Allowed region %	1.5	1.5
Outliers %	0.0	0.0

a Numbers in parentheses are for the highest resolution shell.

b Ramachandran statistics were calculated using MolProbity [37].

## Results and Discussion

### Quinone Substrate Specificity Profiles

**Figure 3:**
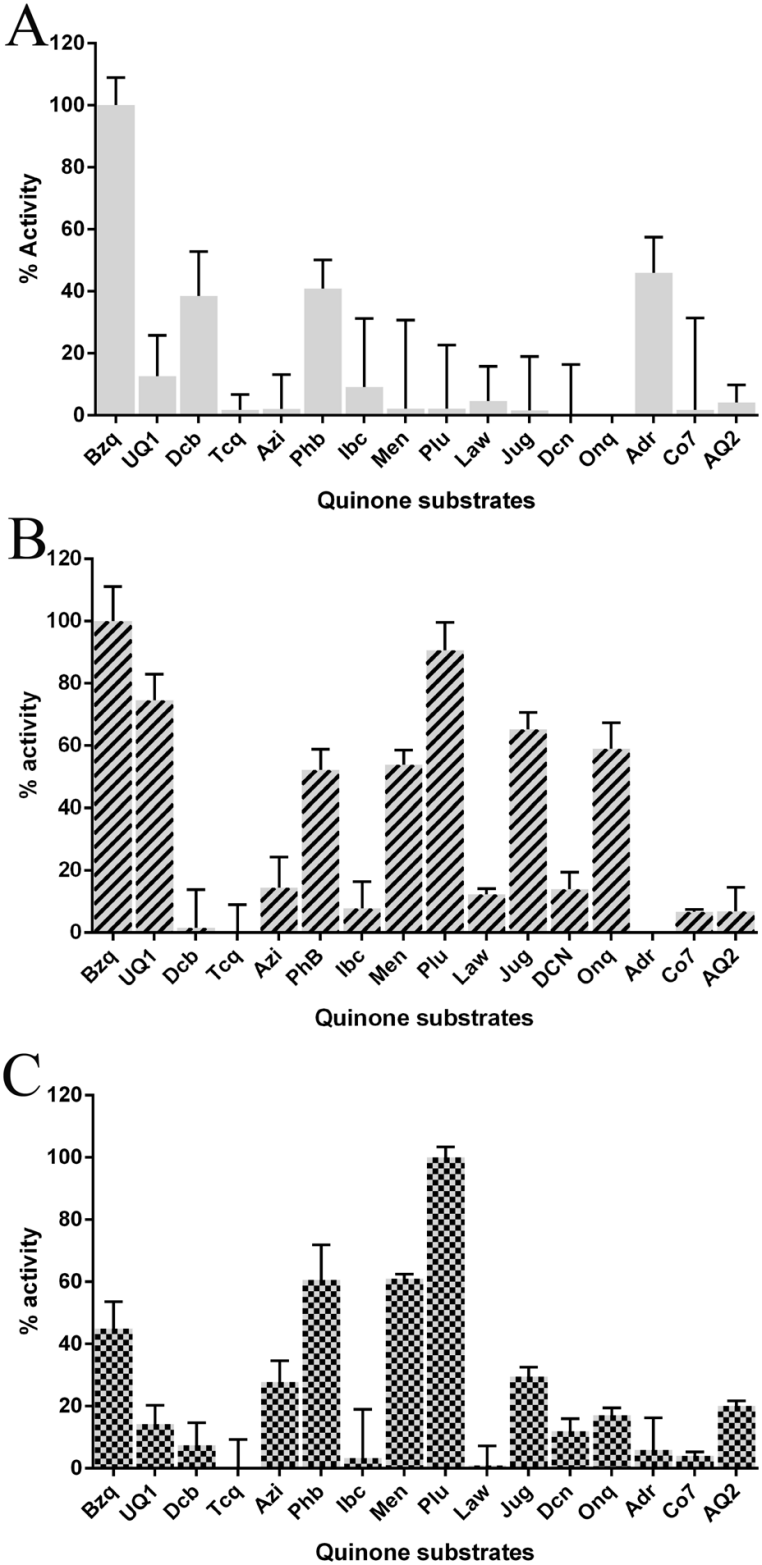
Quinone substrate specificity profiles of paAzoR1 (A), paAzoR2 (B) and paAzoR3 (C). All rates are normalised relative to the maximum rate of NADPH (paAzoR1) or NADH (paAzoR2/paAzoR3) oxidation observed for that enzyme. These maximum rates were as follows: paAzoR1 and paAzoR2 reducing Bzq 0.57 mM.s^−1^.mg of enzyme^−1^ and 7.78 mM.s^−1^.mg of enzyme^−1^ respectively and paAzoR3 reducing Plu 16.8 mM.s^−1^.mg of enzyme^−1^. All rates represent the average of three measurements ± standard deviation.

The substrate specificity profiles of paAzoR1, paAzoR2 and paAzoR3 are shown in Figure 3. Each azoreductase has a distinct substrate specificity profile. The quinones/quinoneimines tested were either benzoquinones or napthoquinones with both ortho and para quinones (Fig. 2). Interestingly paAzoR3 has a quinone reductase activity (Plu –16.8 mM.s^−1^.mg protein^−1^) ∼30 fold higher than that of paAzoR1 (Bzq –0.57 mM.s^−1^.mg protein^−1^). Similarly, the highest activity for paAzoR2 is ∼13 fold higher (Bzq –7.78 mM.s^−1^.mg protein^−1^) than that of paAzoR1. Rates of quinone reduction in all cases were up to two orders of magnitude higher than were observed for reduction of azo substrates as the maximum rate observed was paAzoR3 reduction of methyl red 91.4 µM.s^−1^.mg of protein^−1^ [15], suggesting the enzymes are better suited for carrying out quinone rather than azo reductions. Determination of the K_M_ and V_max_ values for quinone reduction were hampered by the poor aqueous solubility of the quinones and the twin limitations on NAD(P)H concentration to remain within the linear range of the plate reader and maintain a greater than 5∶1 molar ratio of NAD(P)H to quinone. We were able to confirm that the specific activities measured for both Bzq (paAzoR1 and paAzoR2) and Plu (paAzoR3) were within the initial linear part of the rate curve These data confirm that all three azoreductases are NAD(P)H quinone oxidoreductases, however they also highlight that the azoreductases are not all equally well adapted for the role. The large differences in quinone reduction rates may be partly due to the redox potential of the FMN group of the three enzymes. In paAzoR2 and paAzoR3 the negative charge imparted to the FMN after reduction by the NAD(P)H is predicted, based upon homology models [15], to be stabilised by interactions with His144 in the case of paAzoR2 and Tyr145 in the case of paAzoR3. In the case of paAzoR2 the interaction with His144 is supported by the recently published native structure of its homologue (ppAzoR) from *P. putida* MET94 (71% identical PDB: 4C0W [38]). In contrast in paAzoR1 there is no such stabilisation as the equivalent residue is Phe150 which has no polar group to interact with FMN.

**Figure 4:**
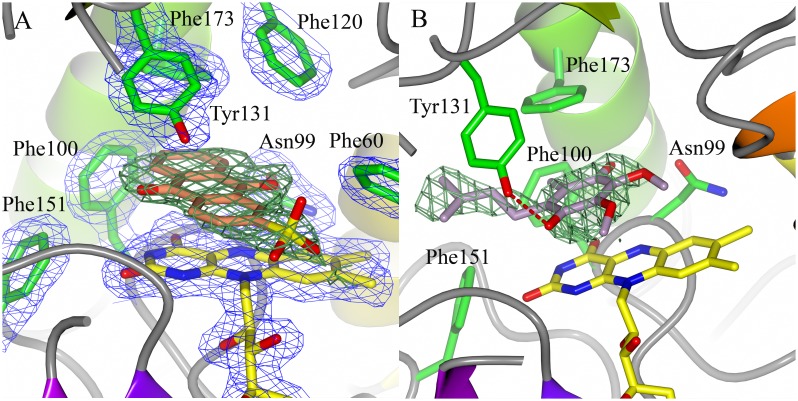
Binding of paAzoR1 to AQN and UQ1. Binding of paAzoR1 to AQN (A) and UQ1 (B). AQN is shown with pink carbon atoms, UQ1 with grey carbon atoms and FMN with yellow carbon atoms. The green mesh is unbiased positive difference density (*F_o_*-*F_c_* map) contoured at 3σ while the blue mesh is the refined 2*F_o_*-*F_c_* map at 1σ. A red dashed line signifies a hydrogen bond. Residues interacting with the ligands are labelled. (A) is based upon PDB 4N65, while (B) is based upon PDB 4N9Q.

paAzoR1 has a preference for benzoquinones *e.g.* Bzq (Fig. 3a). In contrast paAzoR3 prefers naphthoquinones *e.g.* Plu (Fig. 3c). paAzoR2 reduces both benzoquinones and naphthoquinones efficiently (Fig. 3b). One contributing factor to the substrate specificity may be structural, paAzoR2 and paAzoR3 are thought to have significantly larger active sites than paAzoR1. The increased size of the active site of paAzoR2 is supported by the native crystal structure of its homologue ppAzoR [38] where the helix bearing Phe60 shifts significantly outwards, away from the FMN, thus increasing the volume of the active site. In the case of paAzoR3, there are no close homologous structures, however a homology model [15] predicts a F60A substitution that would significantly increase the size of the active site. A substitution at this position was also shown to affect the substrate specificity of AzoR from *E. coli* [39]. The larger size of the active site results in paAzoR3 being able to accommodate significantly larger quinone groups in an orientation similar to that observed for AQN binding to paAzoR1 (Fig. 4a). This data shows that NAD(P)H quinone oxidoreductases from the same organism have complimentary substrate specificity profiles. This had been hinted at by previous ad hoc studies of individual enzymes from *E. coli* (Table 2). This substrate specificity data is also consistent with the results from an earlier study on azoreduction (Fig. S2 [15]).

**Table 2:** Structural and functional information on all azoreductase homologues.

Protein	Structure^a^	Enzymatic activities		
		Azoreductase	NAD(P)H quinone oxidoreductase	Nitroreductase
paAzoR1	2V9C	[21]	This paper	[54]
paAzoR2	ND	[15]	This paper	ND
paAzoR3	ND	[15]	This paper	ND
PA0949	1ZWL	ND	[75]	ND
PA1204	1X77	ND	ND	ND
ecAzoR	2Z98	[8]	[8]	[83]
ecMdaB	2B3D	ND	[66]	ND
ecWrbA	2R96	ND	[74]	ND
ecYieF	3SVL	ND	[84]	ND
ArsH	2Q62	[58]	ND	ND
rsAzoR	ND	[85]	[86]	ND
bsAzoR	1NNI	[13]	[13]	ND
hNQO1	1QBG	[11]	[11]	[11]
hNQO2	2QR2	[11]	[11]	[11]
pnNQO	3U7R	ND	[87]	ND
atNQO	ND	ND	[70]	ND
afNQO	ND	ND	[74]	ND

a PDB codes for structures, ND not determined. Where particular enzymatic activities have been observed a reference is provided, otherwise no reference to the activity has been reported.

Another factor to consider is the redox potential of the quinones, as a previous study has shown that there is a linear relationship between the redox potential of nitroaromatic compounds and their rate of reduction by rat NQO1 [40]. Quinones have a range of redox potentials, Bzq has a redox potential of +0.696 V, while AQN has a redox potential of +0.187 V [41]. The relatively high redox potential of Bzq may explain why all three enzymes reduce Bzq rapidly while the low redox potential of AQN may explain why it is a relatively poor substrate for all three. The position as well as presence or absence of electron withdrawing and donating groups also changes the redox potential of quinones (e.g. Jug has a redox potential of +0.452 V while Law has a redox potential of +0.362 V [41]) and may explain some of the large differences observed between structurally related quinones e.g. Jug and Law in the case of both paAzoR2 and paAzoR3 (Fig. 3b and 3c).

Co7, was recently identified as an inhibitor of human arylamine N-acetyltransferase 1 [42]. This data would be of potential importance were Co7 to be developed as a therapeutic for breast cancer [43].

### Implications of Quinone Reduction

Pseudomonads are unusual in being able to colonise a wide range of hosts from amoeba to humans. Toxic quinones form an integral part of the innate defence of a range of organisms including plants, funghi and invertebrates. Several organisms produce quinones in response to *Pseudomonas* infection via the oxidation of catechols by PPO. Studies have shown the importance of PPO in plant defence against infection by pathogenic *Pseudomonas syringae* [23], [44]. Non-pathogenic *Pseudomonas fluorescens* has also been shown to induce PPO expression in rice plants [45]. The fungus *Agaricus bisporus* up-regulates PPO expression in response to *Pseudomonas tolaasii* infection [46] and in arthropods there is also a significant up-regulation of pro-phenoloxidase in response to infection by *P. aeruginosa* [24]. These results indicate quinone production is an important response to infection by pseudomonads by many hosts.

Humans produce melanin primarily via polymerisation of DOPA-quinone, in contrast plant PPOs can oxidise a range of polyphenols to quinones [47]. We propose the diversity of quinones that are produced in turn drives evolutionary pressure for bacteria to have a range of NAD(P)H quinone oxidoreductases. Plu for example is a potent antibacterial quinone secreted by black walnut trees, that is known to have poor potency against *P. aeruginosa* [48]. Plu is the preferred substrate of paAzoR2 and paAzoR3 (Fig. 3). *P. fluorescens* a close relative to *P. aeruginosa* that has homologues of paAzoR1–3 (Table 3) is insensitive to Jug [49] which is rapidly reduced by paAzoR2 (Fig. 3b).

**Table 3:** Distribution of azoreductase-like enzymes across *Pseudomonas* species.

Gene	*P. fluorescens* a	*P. putida* b	*P. stutzeri* c	*P. denitrificans* d	*P. aureofaciens* e	*P. syringae* pv. tomato^f^
pa0785^g^	78	-	-	68	77	-
pa0949	84	83	86	92	81	81
pa1204	-	-	-	-	-	-
pa1224	-	45	76	-	75	-
pa1225	68	-	-	-	72	-
pa1962^h^	67	71	-	76	67	63
pa2280	83	87 & 80	80	-	82	-
pa2580	79	86	76	81	75	-
pa3223^i^	69	67	-	74	66	-
pa4975	-	78	-	82	-	-

All values are given as percentage identity to the protein from *P. aeruginosa* PAO1. Homologues are defined as having >45% sequence identity based upon Blastp alignments. The strains whose genomes searched are as follows:

a pf0–1,

b KT2440,

c ATCC 17588,

d ATCC 13867,

e 30–84 and ^f^DC3000.

The genes encoding paAzoR1, ^g^paAzoR2^h^ and paAzoR3^i^ are marked.

The hypothesis that the number of azoreductases is related to the host range is supported by data showing Pseudomonads that infect a small range of hosts have fewer azoreductases than *P. aeruginosa* for example *P. syringae pv. tomato*, a pathogen infecting tomato and *A.rabidopsis thaliana*, has two azoreductase-like genes (Table 3) in its genome [50]. A more comprehensive bioinformatics study of azoreductase distribution in pathogenic and non-pathogenic species, alongside further work with gene deletion mutants, will need to be performed in order to test this hypothesis.

*P. aeruginosa* also produces water soluble melanins with benzoquinone functional groups [51] which improve the infectivity of the bacterium [52]. During degradation of phenylalanine and tyrosine, Pseudomonads generate the quinol homogentisate (Hom [53]). Hom can be readily oxidised to a reactive quinone that polymerises to generate melanin [52]. The bacteria needs to maintain Hom in its quinol form prior to secretion to prevent intracellular polymerisation, hence a requirement for NAD(P)H quinone oxidoreductases. All three azoreductases are able to reduce a range of benzoquinones (Fig. 3) hence they would also reduce the oxidised form of Hom.

### Structures of paAzoR1 Binding UQ1 and AQN

In order to help understand the mechanism of quinone reduction by azoreductases the structures of paAzoR1 bound to two quinone substrates (UQ1 and AQN) have been solved via X-ray crystallography. The structure of paAzoR1 bound to AQN was solved at a resolution of 1.8 Å with final *R_free_* and *R_work_* values of 18.5% and 16.2% respectively, while UQ1 was solved at 2.0 Å with final *R_free_* and *R_work_* values of 23.6% and 18.5% respectively (Table 1). No significant conformational changes were observed upon binding of either UQ1 or AQN to paAzoR1 (RMSD 0.22 Å compared to PDB: 2V9C). In both structures, the quinone is only observed binding to one of the two active sites, although positive difference density is observed in the alternate active sites there was insufficient to unambiguously build in the model. During the refinement of the structure of paAzoR1 bound to UQ1, the occupancy of the UQ1 molecule was reduced to 0.5 suggesting that UQ1 was not present in all paAzoR1 molecules.

In the structure of paAzoR1 binding to AQN, AQN makes hydrophobic contacts with Phe60, Phe100, Phe120, Tyr131 and Phe173 (Fig. 4a). AQN also forms π-π stacking interactions with the central ring of FMN while the sulphonate group points into a water filled channel. Neither quinone oxygen is in a position to form a hydrogen bond with Asn99 upon reduction as has been previously observed [54] most probably due to the bulky nature of AQN. Like AQN, UQ1 forms π-π stacking interactions with FMN and makes hydrophobic contacts with Phe100, Phe120, Tyr131 and Phe173 (Fig. 4b) but it also hydrogen bonds with Tyr131 via the quinone oxygen and forms extra hydrophobic contacts with Phe151.

The binding orientations of UQ1 and AQN differ significantly, with UQ1 binding parallel to the ring system of FMN while AQN binds at almost a 90° angle to FMN (Fig. 4). These differing binding orientations correspond to those observed when methyl red binds to either the wild type enzyme [21] in the case of AQN and the Y131F mutant [14] in the case of UQ1. The binding orientation of UQ1 is also consistent with the binding of both balsalazide and nitrofurazone to the wild type enzyme [15], [54]. The binding orientation of AQN to paAzoR1 is consistent with binding of menadione to hNQO2 (PDB 2QR2). A recent X-ray crystal structure shows the binding of AQN to the ppAzoR (PDB: 4C0X [38]). AQN is shown to bind in an orientation similar to that observed for UQ1 bound to paAzoR1, with the sulphonate positioned in solvent outside the pocket. In contrast to the structure with paAzoR1, AQN is accommodated inside the active site of ppAzoR via a number of significant side chain conformational shifts. Due to the altered binding conformation of AQN, compared to the paAzoR1 structure, a water bridged hydrogen bond can form between AQN and Asn97 (Asn99 in paAzoR1). This water is similar to that seen in the structure of paAzoR1 bound to nitrofurazone [54] and is likely to be involved in the mechanism of reduction.

In the case of UQ1 a δ^+^ methoxy substituted carbon of the quinone ring is 3.5 Å from the N5 of FMN making it an ideal candidate for hydride transfer which corroborates the previously proposed mechanism of quinone reduction based upon comparison with azo reduction [16]. In the case of AQN one would expect the δ^+^ carbon, which accepts the hydride, to be the carbon attached to the negatively charged sulphate. However the sulphate of AQN is not easily accommodated within the primarily hydrophobic active site of paAzoR1. As a result an unmodified carbon atom (Fig. S3) accepts the proton in a less favourable reaction possibly contributing to the lower rate of reduction observed of AQN by all azoreductases compared to other quinones (Fig. 3).

### Identification of the Extended Azoreductase Family of Enzymes

**Figure 5:**
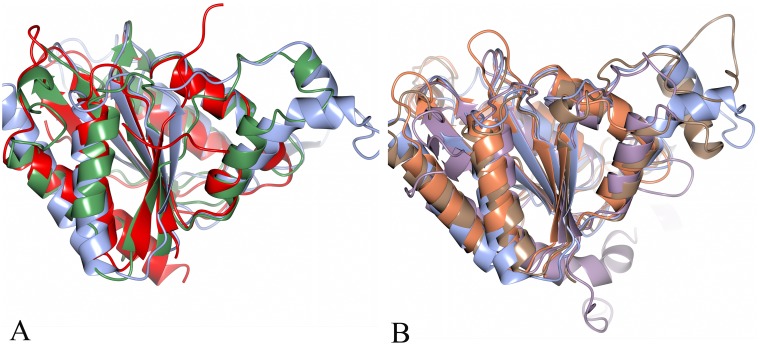
Structures of azoreductase homologues overlaid onto paAzoR1. (A) Structures of ArsH (PDB: 2Q62 - lilac), ecMdaB (PDB: 2B3D – coral) and hNQO2 (PDB: 2QR2 - brown) overlaid onto paAzoR1. (B) Structures of PA0949 (PDB: 1ZWL - red) and PA1204 (PDB: 1×77 - green) overlaid onto paAzoR1. In all cases, one monomer of the dimer is shown. In both cases, paAzoR1 is in blue (PDB: 2V9C). All alignments were carried out via secondary structure matching and images were generated in CCP4MG [82].

The availability of the sequence of the *P. aeruginosa* PAO1 genome [26] allowed initial identification of paAzoR1, paAzoR2 and paAzoR3 via sequence homology to *E. coli* AzoR [21] using a sequence identity cut-off of 30%. Literature reports show that proteins with azoreductase and NAD(P)H quinone oxidoreductase activities have highly diverse amino acid sequences (Fig. 1). Due to their conserved structure (Fig. 5) and mechanism of action [16] we propose that the azoreductases and NAD(P)H quinone oxidoreductases form an enzyme superfamily. This sequence diversity stems from the fact that all reactions occur on the flavin cofactor (either FMN or FAD) which is anchored to the protein via a large number of sequence independent interactions with the protein backbone [15]. Due to the sequence diversity several putative azoreductases were missed during the original bioinformatics scan. The following genes are proposed to encode azoreductases: *pa0949*, *pa1204*, *pa1224*, *pa1225*, *pa2280*, *pa2580* and *pa4975*, based upon sequence homology to proteins of known structure and function. The phylogenetic tree that illustrates their evolutionary relationship to each other and to characterised enzymes from other species is shown in Figure 1, the available structural and functional data for each characterised enzyme in the tree is displayed in Table 2.

#### Homologues of known azoreductases

The homologues of four putative azoreductase genes from *P. aeruginosa* have been characterised as being able to reduce azo dyes. The first of these is the protein encoded by *pa2280* which is a member of the ArsH enzyme family. ArsH was initially identified as part of the arsenic resistance operon of *Yersiniae enterocolitica* [55] although gene deletion does not affect resistance to arsenic. A functional arsenic resistance operon was identified in *P. aeruginosa* PAO1 [56] consisting of ArsR, ArsB and ArsC (PA2277–PA2279) and *pa2280* is predicted by DOOR [57] to be part of the same operon as these genes. ArsH from both *Sinorhizobium meliloti* (82% similarity [58]) and *Synechocystis* sp PCC 6803 (78% similarity [59]) have been shown to reduce azo dyes. *Synechocystis* sp PCC 6803 ArsH also reduces quinones [59]. The structure of ArsH from *S. meliloti* [58] has the same overall core fold as paAzoR1 (RMSD = 2.2 Å – Fig. 5a). The major difference in structure between paAzoR1 and the ArsH family is a C-terminal extension of around 30 amino acids that is absent from most other members of the azoreductase family. The function of this C-terminal extension is unclear.

The second putative azoreductase in this class is encoded by *pa4975*. The gene product of *pa4975* is part of the arginine transaminase operon of *P. aeruginosa* [60] although its function within this operon remains unclear. PA4975 is unusual in bacteria in that it shows significant sequence similarity to the human enzyme NQO2 (61% similarity). hNQO2 regulates proteasomal degradation of a range of proteins [61], and also regulates TNFα induced apoptosis [62]. hNQO2 can reduce both azo and quinone substrates [11]. The structure of hNQO2 has been solved and has the same overall fold as paAzoR1 (RMSD = 1.5 Å – Fig. 5a).

The third and fourth azoreductase-like enzymes are encoded by *pa1224* and *pa1225* which show sequence homology to the human azoreductases hNQO1 (54% similarity) and hNQO2 (48% similarity) respectively. hNQO1 also known as DT-diaphorase, regulates proteasomal degradation of various proteins including transcription factors such as p53 and C/EBPα [63]. Although adjacent on the chromosome the proteins are encoded on opposite strands and hence are unlikely to be co-regulated and little is known of the function of either gene. hNQO1, like hNQO2, is both an azo and quinone reductase [11]. hNQO1 shares the same overall fold as paAzoR1 (RMSD 2.1 Å [64]) but like ArsH has a long C-terminal extension.

#### Homologues of known NAD(P)H quinone oxidoreductases

Close homologues of several other *P. aeruginosa* genes have an NAD(P)H quinone oxidoreductase activity and the same overall fold as paAzoR1 but have yet to be tested for azoreductase activity. The protein encoded by *pa2580* shares significant homology with a group of enzymes known as MdaB. The MdaB family was initially identified in *E. coli* due to its overexpression causing increased doxorubicin resistance [65]. The structure of *E. coli* MdaB (75% similarity) has been solved and has the same overall fold as paAzoR1 (RMSD = 2.2 Å – Fig. 5a). MdaB from both *E. coli* [66] and *Helicobacter pylori* (71% similarity [67]) have NAD(P)H quinone oxidoreductase activities.

The protein encoded by *pa1204* is part of the BexR regulon in *P. aeruginosa* [68] and is part of an operon consisting of *pa1202*–*pa1205* that is strongly up-regulated in BexR gene (*pa2432*) deletion mutants. The structure of PA1204 has been solved and was shown to have the same overall fold as paAzoR1 (RMSD = 2.3 Å -Fig. 5b [69]). The closest characterised homologue of PA1204 is an enzyme from *A. thaliana* that shows NAD(P)H quinone oxidoreductase activity (69% similarity [70]).

The protein encoded by *pa0949* is part of the WrbA family, named for their strong interaction with tryptophan repressor [71]. In *P. aeruginosa* as in many bacteria, expression of PA0949 is controlled by the PhoP/PhoQ two component regulatory system [72]. The structure of paWrbA has been solved and shows the same overall fold as paAzoR1 (RMSD = 2.3 Å - Fig. 5b [73]). The *E. coli* homologue of WrbA (56% similarity) has NAD(P)H quinone oxidoreductase activity [74]. Although phylogenetic analysis does not support a common ancestor of the azoreductase and WrbA enzyme families (Fig. 1), we believe the structural and enzymatic data is sufficient for it to be included as a likely candidate to be an azoreductase.

Recent data have shown that PA0949, PA1204, PA2580 and PA4975, have NAD(P)H quinone oxidoreductase activities [75]. This data supports their inclusion in this list of possible azoreductases.

#### Distribution of azoreductase like genes in *Pseudomonas* species

It is interesting to note that not all Pseudomonads have the same complement of azoreductases that are observed in *P. aeruginosa* PAO1 (Table 3). A range of genomes from both fluorescent and non-fluorescent *Pseudomonas* species were searched for proteins that were close homologues (>45% sequence identity) of those found in *P. aeruginosa* PAO1. In the six genomes that were selected only one putative azoreductase (paWrbA or *pa0949*) was present in all. WrbA homologues are also found in a range of other bacteria including *E. coli* (Table 2) however WrbA is not essential for survival [74]. In contrast, close homologues of PA1204 were absent from all six genomes. Interestingly the closest homologue of PA1204 in *P. fluorescens* pf0–1 (Pfl01_2192-38% identical) does have homologues in *P. putida* (69% identical), *P. denitrificans* (74% identical), *P. aureofaciens* (86% identical) and *P. syringae* pv tomato (75% identical).

## Conclusions

Azoreductases are a varied family of enzymes that have been identified in almost all species (with the exception of viruses). Their ability to reduce a wide variety of both endogenous and exogenous compounds has complicated the identification of their physiological substrate and hence their function. The quinones identified in this study appear to be the best substrates for the enzymes yet identified, indicating that they may act as the physiological substrate for these enzymes. Enzymes with a flavodoxin-like fold are frequently observed to have both azoreductase and NAD(P)H quinone oxidoreductase activities (Table 2) therefore we suggest that these activities are indistinguishable in these enzymes.

Water soluble quinones are an important part of the defence mechanism of many organisms against bacteria including *P. aeruginosa*. In order to detoxify these quinones *P. aeruginosa* must reduce the quinone to the more stable quinol. Plants in particular are able to oxidise a range of catechols to quinones via their PPO enzymes, and as a result Pseudomonads need to be able to reduce a range of different quinones in order to survive in plant systems. We have shown the azoreductase family are very active NAD(P)H quinone oxidoreductases with broad specificity profiles and as a result they may play an important role in detoxifying the quinones secreted by many of the host organisms of *P. aeruginosa*. Infection of model plants with azoreductase gene deletion mutants in *P. aeruginosa* could be studied to improve our understanding of the role of azoreductases in plant infection.

In addition to their enzymatic activities a number of azoreductase homologues have been shown to alter the action of proteins via mechanisms independent of their catalytic function. The human azoreductases hNQO1 and hNQO2 regulate ubiquitin-independent proteasomal degradation of various proteins via direct protein-protein interactions [61], [63], while in *E. coli* the NAD(P)H quinone oxidoreductase KefF [76] interacts with the potassium channel KefC and regulates its ion flux [77]. The importance of these protein-protein interactions in the physiological function of azoreductases requires further detailed study.

## Supporting Information

Figure S1 **Flowchart outlining the bioinformatics procedure applied to identifying members of the azoreductase family.**
(PDF)

Figure S2 **Azo compound substrate specificity profiles of paAzoR1 (A), paAzoR2 (B) and paAzoR3 (C).** Abbreviations for all substrates are as follows; balsa – balsalazide, pabsa - p-aminoazobenzene-4′-sulfonate, sulfa – sulfasalazine, olsa – olsalazine, metred – methyl red, tropa- tropaeolin O, amar – amaranth, ponc S/BS – ponceau S/BS,. All rates are normalised relative to the maximum rate of reduction of Balsalazide (paAzoR1 –29.7 µM.s^−1^.mg^−1^), ponceau BS (paAzoR2 –26.3 µM.s^−1^.mg^−1^) or methyl red (paAzoR3 –91.4 µM.s^−1^.mg^−1^). All rates represent the average of three measurements with error bars representing ±standard deviation from three replicates. Data is taken from [15].
(TIF)

Figure S3 **Mechanism of AQN reduction by paAzoR1.**
(TIF)
