# Supplementary figures and images for: Identification of NAD(P)H Quinone Oxidoreductase Activity in Azoreductases from P. aeruginosa: Azoreductases and NAD(P)H Quinone Oxidoreductases Belong to the Same FMN-Dependent Superfamily of Enzymes
Source: PLoS One. 2014 Jun 10;9(6):e98551. doi: 10.1371/journal.pone.0098551 (PMC4051601; doi:10.1371/journal.pone.0098551)

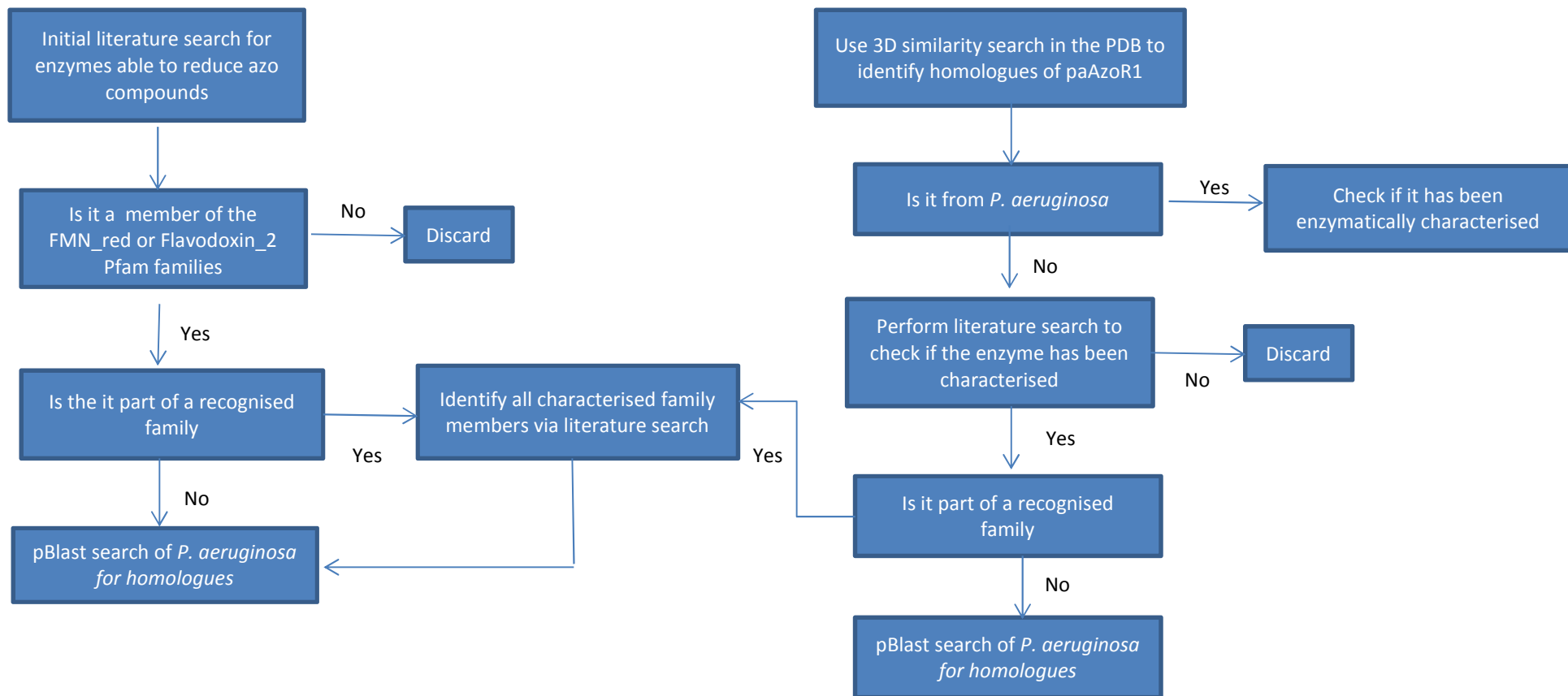

Supplement: Figure S1 — Flowchart outlining the bioinformatics procedure applied to identifying members of the azoreductase family. (PDF) [file pone.0098551.s001.pdf]

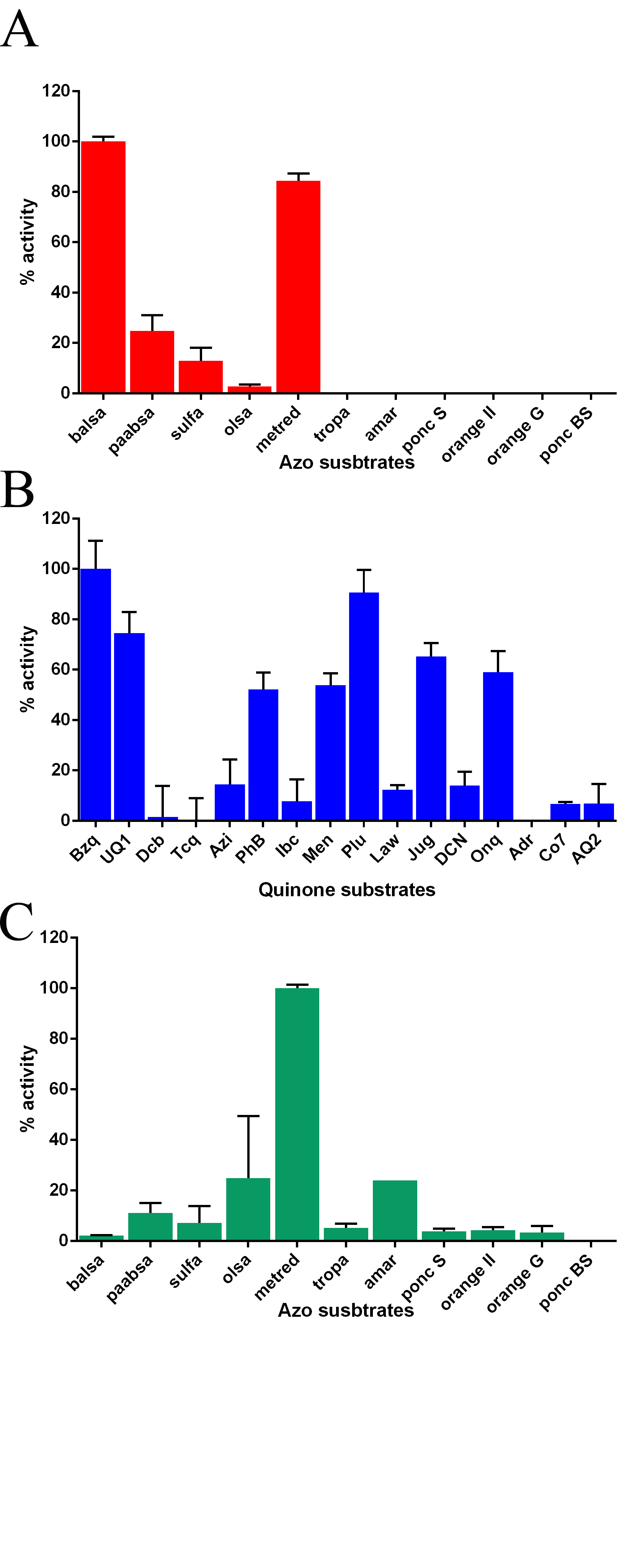

Supplement: Figure S2 — Azo compound substrate specificity profiles of paAzoR1 (A), paAzoR2 (B) and paAzoR3 (C). Abbreviations for all substrates are as follows; balsa – balsalazide, pabsa - p-aminoazobenzene-4′-sulfonate, sulfa – sulfasalazine, olsa – olsalazine, metred – methyl red, tropa- tropaeolin O, amar – amaranth, ponc S/BS – ponceau S/BS,. All rates are normalised relative to the maximum rate of reduction of Balsalazide (paAzoR1 –29.7 µM.s−1.mg−1), ponceau BS (paAzoR2 –26.3 µM.s−1.mg−1) or methyl red (paAzoR3 –91.4 µM.s−1.mg−1). All rates represent the average of three measurements with error bars representing ±standard deviation from three replicates. Data is taken from [15]. (TIF) [file pone.0098551.s002.tif]
